# Central Auditory Plasticity from Molecules to Behavior

**DOI:** 10.3390/brainsci11050573

**Published:** 2021-04-29

**Authors:** Maria E. Rubio

**Affiliations:** Departments of Neurobiology and Otolaryngology, University of Pittsburgh Medical School, BST3 Building, 3501 Fifth Avenue #10016, Pittsburgh, PA 15261, USA; mer@pitt.edu; Tel.: +1-412-624-6939

Understanding how, when, and for how long the adult central auditory system adapts to hearing loss and aging is an important topic that is currently studied across the globe. Central auditory plasticity can occur from very rapid adaptation to long-term changes, and varies depending on the history of the sensory input or the influence of attention, learning, and decision-making. Despite continuous efforts, it is still unknown whether central auditory plasticity to hearing loss represents homeostatic mechanisms or maladaptive changes that lead to tinnitus and hyperacusis. Studies in this Special Issue of “Central Auditory Plasticity” encompass research relevant to the advancement of our understanding of the neurobiology underlying structural, molecular, electrophysiological and behavioral adaptations to hearing loss and ageing.

The study by Kurioka et al. [1] focused on the potential reversibility and differential levels of expression of vesicular glutamate transporters 1 and 2 (VGLUT-1 and -2) after conductive hearing loss. Findings aim to contribute to the understanding of the mechanisms of auditory-dependent plasticity in response to hearing loss. In the study by Poveda and colleagues [2], authors investigated the short- and long-term changes in Kv1.1 and Kv3.1b expression after peripheral deafferentation in two different nuclei of the central auditory pathway, the cochlear nucleus and the inferior colliculus. The results reveal that post-lesion adaptations do not necessarily involve stereotyped plastic mechanisms along the entire auditory pathway. The generation of phantom sounds (tinnitus) can result from noise exposure; however, understanding of its underlying mechanisms are limited. The review by Wang et al. [3] focuses on the assessment of noise-induced hearing loss, available treatments, and development of new pharmacologic and non-pharmacologic treatments based on recent studies of central auditory plasticity and adaptive changes in hearing. Results of animal and human studies are essential to improve our understanding of neural mechanisms underlying the generation of noise-induced hearing loss, and are critical for developing effective treatment and therapies.

Aging-related structural, molecular and functional changes occur at every level of the central auditory system. One of the most common findings is a loss of synaptic inhibition with aging, which has been proposed to be the base of aging-related changes in auditory cognition, such as diminished speech perception in complex environments and the perception of tinnitus. Studies speculated that downregulation of synaptic inhibition is a consequence of peripheral deafferentation and, therefore, is a homeostatic mechanism to restore excitatory/inhibitory balance. The study by Wang and colleagues [4] shows that a reduced activity in aged D-stellate neurons decreases the overall inhibition and enhances the central gain in the cochlear nucleus, which serves as a compensatory mechanism to counteract the reduced sensory inputs from the auditory periphery during age-related hearing loss. Further studies are needed to determine whether disinhibition represents a form of compensatory plasticity or in contrast, is a form of maladaptive plasticity. In Ibrahim and Llano [5], the authors provide a new perspective on how aging-related disinhibition may, in part, be related to the high metabolic demands of inhibitory neurons relative to their excitatory counterparts. Understanding the relative importance of these mechanisms will be critical for the development of treatments for the underlying causes of aging-related central disinhibition.

In order to accurately localize sounds on the horizontal plane, the binaural hearing system relies on computing the interaural time and level differences from the incoming acoustic signal. Improving binaural hearing has become highly relevant in clinical settings due to the appreciation of deaf patients’ needs to communicate and function in realistic, complex auditory environments. The study by Thakkar and colleagues [6] aimed to unrevealed how age-related, acoustic, and electric experience impact binaural sensitivity in adults with bilateral cochlear implants. Individuals with unilateral cochlear implant have difficulty with pitch perception, and adding a hearing aid in the non-implanted ear is potentially beneficial. In Zhang et al. [7], authors provide evidence for the practice of fitting a hearing aid in the non-implanted ear to take advantage of the potential bimodal benefit, to facilitate speech learning in kindergarten-aged children with a unilateral cochlear implant.

Prepulse inhibition (PPI) implies plasticity of a reflex and is related to automatic or attentional processes, and might be considered a potential marker of short- and long-term plasticity. The PPI plays a key role in normal brain activity; however, little is known about the intimate physiology, circuitry, and neurochemistry of sensorimotor gating mechanisms. In Gómez-Nieto et al. [8], authors review the current literature, focusing on studies questioning the neuroanatomy, connectomics, neurotransmitter–receptor functions, and sex-derived differences in the PPI process during normal brain function and in neuropsychiatric disorders. Hearing loss and social isolation are factors that independently influence social behavior. In human subjects, hearing loss may also contribute to objective and subjective measures of social isolation. Although the behavioral relationship between hearing loss and social isolation is evident, there is little understanding of their interdependence at the level of neural systems. Keesom and Hurley [9] review the interactions of the serotonergic pathways within the auditory system as well as within other brain regions. Such interactions are difficult to tease apart, but they start providing strong evidence to further explore the serotonergic pathways as potential intervening mechanisms between the related conditions of hearing loss and social isolation, and the affective and cognitive dysfunctions that follow psychiatric disorders.
